# Acceptability of an Intervention to Prevent Older Adult Mistreatment Among Family Caregivers to Persons With Dementia: Multimethod Pilot Study

**DOI:** 10.2196/73778

**Published:** 2025-07-30

**Authors:** Kylie Meyer, Wenxing Wei, Jeanine Yonashiro-Cho, Susanna Mage, Sohee Kim, Elliane Irani, Christopher Burant, Zachary Gassoumis, Erin Gentry Lamb, Jaclene A Zauszniewski, Donna Benton

**Affiliations:** 1 Frances Payne Bolton School of Nursing Case Western Reserve University Cleveland, OH United States; 2 Jack, Joseph, and Morton Mandel School of Applied Social Sciences Case Western Reserve University Cleveland, OH United States; 3 Keck School of Medicine University of Southern California Los Angeles, CA United States; 4 Leonard Davis School of Gerontology University of Southern California Los Angeles, CA United States; 5 Department of Bioethics School of Medicine Case Western Reserve University Cleveland, OH United States

**Keywords:** family caregiving, dementia, elder mistreatment, intervention, acceptability, qualitative

## Abstract

**Background:**

Older adult mistreatment occurs in many as one-half of dementia care partners. Psychological mistreatment is the most common form of older adult mistreatment by family caregivers and is known to create mental health morbidities among care recipients. The Knowledge and Interpersonal Skills to Develop Enhanced Relationships (KINDER) intervention is among the first older adult mistreatment prevention interventions focused on family caregivers. KINDER was designed to prevent psychological mistreatment of older adults. Caregivers found the initial asynchronous web-based version (KINDER 1.0) to be acceptable but expressed a desire to engage with other family caregivers. KINDER was revised to integrate 3 facilitated small group discussion sessions conducted by videoconference. This study examines the acceptability of a revised KINDER intervention. This research addresses the extent to which caregivers find a novel approach to older adult mistreatment prevention to be acceptable.

**Objective:**

This study aims to evaluate the acceptability of the revised *KINDER* intervention.

**Methods:**

The investigators conducted semistructured qualitative interviews with a purposive sample of family caregivers following participation in *KINDER* (N=11) and collected postintervention survey data (N=71). The qualitative interview codebook and survey questions were informed by the Theoretical Framework of Acceptability by Sekhon et al. Components of acceptability in this framework include affective attitude, burden, ethicality, intervention coherence, opportunity costs, perceived effectiveness, and self-efficacy at completing activities. Qualitative interviews were coded by 2 independent coders using a thematic analytic approach. Survey data were analyzed using frequencies and percentages.

**Results:**

Of the 98 caregivers who attended *KINDER,* 71 (72%) completed satisfaction surveys. Caregivers reported high levels of overall satisfaction with *KINDER*; 80% (53/66) of participants reported they were “Very Satisfied” with the intervention, and 20% (13/66) indicated they were “Satisfied.” More than 80% of caregivers (56/69, 81%) rated the newly added group discussions as being “Very valuable.” Qualitative findings supported positive attitudes revealed in survey responses. Themes addressed (1) the interventions’ alignment with caregiver values (affective attitude, intervention coherence, ethicality), (2) beliefs about the effectiveness of the program (perceived effectiveness), (3) difficulty participating in the program relative to its perceived overall value (burden, opportunity cost, self-efficacy), and (4) recommendations to further improve the intervention.

**Conclusions:**

These findings indicate that *KINDER* was well received among family caregivers, who reported high levels of satisfaction and positive feedback on its components. The addition of virtual group discussion sessions was particularly valued. The use of multiple data collection methods in this research provided a comprehensive understanding of caregiver experiences. This study contributes to current knowledge by demonstrating the acceptability of a novel intervention to prevent older adult mistreatment by family caregivers to persons with dementia. Future research should focus on testing the efficacy of *KINDER* and exploring its implementation in health and social service settings.

**Trial Registration:**

ClinicalTrials.gov NCT05783102; https://clinicaltrials.gov/study/NCT05783102

## Introduction

### Background

Older adult mistreatment is defined by the World Health Organization (WHO) as “a single or repeated act, or lack of appropriate action, occurring within any relationship where there is an expectation of trust, which causes harm or distress to an older person” [1]. Among community-dwelling older adults with dementia, 80% receive informal care at home from family caregivers [2]. Despite providing essential care, caregivers, unfortunately, are among the most likely to engage in older adult mistreatment: An estimated one-third to over one-half of caregivers self-report mistreatment toward the care recipient with dementia [3,4]. Given the high prevalence of mistreatment of older adults by caregivers to persons with dementia, this population is an important group to target in interventions designed to prevent older adult mistreatment. However, little is known about evidence-based approaches to prevent older adult mistreatment by family caregivers, let alone the acceptability of such approaches to potential users. This study considers the acceptability of a psychoeducational intervention to prevent psychological mistreatment of older adults in dementia care contexts.

Psychological mistreatment of older adults accounts for the majority of mistreatment by caregivers and is the most common type of older adult mistreatment outside neglect [5,6]. Behaviors characterizing psychological mistreatment of older adults include verbal and nonverbal behaviors that may inflict emotional pain, fear, or distress in the older person, including actions like making threats, humiliating, or ignoring the older person [7]. Victims of psychological older adult mistreatment face an increased risk of anxiety, depression, and chronic disease [8,9]. Importantly, psychological mistreatment of older adults often co-occurs with other types of mistreatment, such as neglect and physical abuse [10]. With the number of persons with Alzheimer disease and related dementias projected to double by 2060, there is an urgent need to identify and develop effective strategies to prevent caregiver psychological mistreatment toward this population [11].

Knowledge and Interpersonal Skills to Develop Enhanced Relationships (*KINDER*) is a psychoeducational intervention developed to prevent psychological mistreatment by caregivers toward the person with dementia for whom they provide care. The intervention focuses on building healthy relationships between caregivers and their care recipients as a framework to prevent mistreatment, following similar successful approaches in the area of intimate partner violence [12,13]. After identifying barriers to participation in its initial pilot study, *KINDER* recently underwent an additional round of testing to determine the intervention’s feasibility, preliminary efficacy, and acceptability [14]. Findings from the second pilot test indicate feasibility, including an 82% retention rate in a pre- and posttest pilot study, as well as preliminary efficacy, including statistically significant decreases in the occurrence of psychological mistreatment of older adults [15]. This study examines the acceptability of the *KINDER* intervention. “Acceptability” is a concept believed to drive adherence, engagement, and future adoption of behavioral intervention and is, therefore, a critical criterion for intervention translation [16].

### Interventions to Prevent Older Adult Mistreatment Among Family Caregivers

Few interventions have been tested to prevent mistreatment by caregivers toward persons with dementia, regardless of mistreatment type [17,18]. Even less is known about the acceptability of these interventions for their intended audiences, which is a critical factor in determining participation adherence, implementation, and outcomes. In one of the earliest efforts to test the impact of an information-based supportive caregiver intervention on reducing older adult mistreatment, the *Strategies for Relatives* (*START*) program sought to mitigate caregiver mistreatment of older adults by addressing known mental health mistreatment risk factors affecting caregivers, such as depression, through a supervised manual-based coping program [19]. Despite positive findings of decreased anxiety and depression for caregivers, results from this study showed no differences between the intervention and control group participants on outcomes related to older adult mistreatment. Recently, the *Comprehensive Older Adult and Caregiver Help* (*COACH*) intervention, also aimed at reducing older adult mistreatment, revealed promising results [20]. Caregivers who participated in up to 12 weekly 1-on-1 supportive caregiver coaching sessions, which included topics like goal setting, experienced a statistically significant decrease in mistreatment behaviors compared with an information-only control group condition. Although such findings are promising for determining efficacious approaches to prevent older adult mistreatment in a care context, neither the *START* nor *COACH* program reported on intervention acceptability.

Similarly, the *RISE* intervention combines tailored support, including goal setting, with advocacy for individuals in the Adult Protective Service (APS) system. Consistent with its person-centered approach, at the discretion of the person who experienced mistreatment, the intervention allows for the inclusion of perpetrators of mistreatment, with goals that could include repairing the relationship [21]. Although acceptability data from the older adult who experienced mistreatment and caregivers involved in *RISE* have not been published, a recent study examining APS worker perceptions of *RISE* found that the program was described as being a valuable resource to address older adults’ needs that fall outside of or otherwise complement what APS can provide [22]. Among caregivers who engage in mistreatment of older adults, little is known about their willingness to participate in interventions like *COACH* and *RISE*. These interventions nonetheless provide important examples of ongoing innovative approaches researchers are taking to mitigate older adult mistreatment.

### The KINDER Intervention

#### KINDER Development

*KINDER* was developed to reduce the occurrence of psychological mistreatment of older adults in the context of family caregiving to persons with dementia by focusing on the relationship between the caregiver and care recipient. The goal of *KINDER* is both the primary *and* secondary prevention of psychological mistreatment of older adults; that is, the aim is 2-fold: to prevent mistreatment from occurring in the first place and reduce such behaviors if they are already occurring [17]. Although the intervention focuses on psychological mistreatment of persons with dementia by a caregiver, the content also addresses other forms of mistreatment, like neglect and physical mistreatment. This is important given the likelihood of more than one type of mistreatment occurring simultaneously within the care dyad [10]. To distinguish between the first and the more recent version of *KINDER*, we refer to the initial version as “*KINDER* 1.0” and the most recent version as “*KINDER*.”

#### KINDER 1.0

##### Structure and Components

*KINDER* 1.0 was tested between March 2020 and September 2021. This first version was an 8-week, web-based intervention [14,23]. The intervention was delivered asynchronously to give caregivers flexibility with completing lessons. Caregivers received weekly reminders to log into the web-based portal to complete independent lessons. Content was developed based on existing literature about risk factors for low-quality care, including potentially harmful care and older adult mistreatment, related to the care relationship (eg, prior occurrence of mistreatment) [24], as well as findings from qualitative focus groups and 1-on-1 interviews with caregivers about their experiences managing relationship strain [25]. Topics included education about community resources and seeking help, self-care, common brain changes with dementia, responding to behavioral symptoms of dementia, recognizing older adult mistreatment, communicating with persons with dementia, managing difficult topics around safety and independence, managing mental health conditions, and coping with stress related to the care relationship. Each lesson in *KINDER* 1.0 included a brief story-based video informed by qualitative focus group findings, a reading that elaborated on topics from videos (eg, communicating with a person with dementia), a reading quiz, and a journal reflection. In addition, caregivers were advised each week to engage in a pleasant, self-care activity.

##### Summary of Findings From KINDER 1.0

Of the 27 caregivers who enrolled in *KINDER* 1.0, only 7 completed the intervention (ie, each lesson through Lesson 8) [14]. Although a low proportion of completers is not unusual for an asynchronously delivered intervention, the investigators sought to better understand low treatment fidelity [26]. In-depth, semistructured qualitative interviews were conducted with these caregivers to understand their experiences while participating in *KINDER* 1.0. Findings illustrated overall acceptability of the program among completers, who reported liking the program’s authenticity and emphasis on self-care. Only one caregiver identified discomfort with the content, noting that she could not relate to depictions of caregivers becoming angry and that this made her feel uneasy. Technical problems with the web-based platform (eg, too complex of a display to track pleasant activities) were also identified as a likely barrier to participation. When asked how to improve the program beyond the technical challenges, caregivers expressed a desire to interact with other family caregivers during their participation in the program.

#### Revisions to KINDER 1.0

Based on caregivers’ feedback on *KINDER* 1.0 and prior studies demonstrating the value of group-based interventions, *KINDER* 1.0 was revised as *KINDER* to include virtual synchronous and group-based components over an 8-week period [27,28]. We integrated 3 group-based discussion sessions held via videoconference into independent lessons. Small group discussion sessions occurred during Week 1, Week 4, and Week 8. Discussion sessions were facilitated by 2 members of the study team, including the intervention developers (a geropsychologist and a gerontologist) or a nurse interventionist who received training prior to the intervention on how to deliver the program with fidelity.

Participants received 2 weekly emails reminding them to complete independent lesson activities. Participants also received a telephone introduction from one of the facilitators prior to the start of the first discussion session (ie, Week 0) and 2 check-in calls during Week 3 and Week 6 to support adherence. Web content from *KINDER* was adapted into a workbook, available as a PDF and as a printed hard copy mailed to participants, depending on the caregiver’s preference. Videos could be accessed by participants using a URL or a QR code found in their workbook. The first figure in the publication by Meyer et al [15] illustrates the structure of the revised intervention.

### Purpose of This Study

This multimethod study aimed to evaluate the acceptability of the revised *KINDER* intervention among caregivers of persons with dementia to support future implementation. Evidence of acceptability is particularly important for the *KINDER* intervention, given the sensitive nature of the topics covered, which could deter future participation among caregivers. To determine intervention acceptability, we administered satisfaction surveys to caregivers following their participation in the intervention and conducted in-depth qualitative interviews with program completers to elucidate multiple components of acceptability and understand opportunities for program improvement. Findings will inform future *KINDER* refinements and guide decisions about whether the intervention should proceed to efficacy testing.

## Methods

### Study Design

This study used a design incorporating posttest data collection only, including collection of qualitative survey data and qualitative interview data. Of the 151 caregivers registered to participate in *KINDER*, 98 caregivers completed the program when it was offered in a community-based service setting from April 2023 to March 2024 [15]. Enrollment in the feasibility study was optional; 72% (71/98) of caregivers who attended the program indicated interest in participating, of which 63% (45/71) were enrolled in a pre- and posttest feasibility study. In the posttest survey, participants were asked about the acceptability of the *KINDER* intervention via survey. Caregivers who were not enrolled in the pre- and posttest study also received an invitation to complete a satisfaction survey at the end of the program. Of the 53 caregivers who participated in *KINDER* outside the feasibility study, 64% (34/53) completed the satisfaction survey. Participants were eligible if they registered for and attended the *KINDER* intervention, which was open to family caregivers to persons with dementia. Although a sample size was identified for the pre- and posttest trial to evaluate preliminary efficacy, no sample size was predetermined for collection of acceptability data. Cohorts were conducted until the pre- and posttest sample size for enrollment was reached.

This study followed the model of feasibility by Bowen et al [29], wherein intervention acceptability is one component of feasibility; “acceptability” is the focus of this study [29]. Additional information on the pre- and posttest study design and additional feasibility data, such as preliminary efficacy, can be found in the study by Meyer et al [15]. Multimedia Appendix 1 illustrates the creation of the analytic sample for survey participants and describes differences in how caregivers received survey invitations, as well as distribution of incentives for caregivers enrolled in the pre- and posttest study and those who completed the *KINDER* program outside the trial.

### Conceptual Model of Acceptability

Data collection tools to assess intervention acceptability were informed by the Theoretical Framework of Acceptability (TFA) by Sekhon et al [30]. This model includes 7 components of acceptability with intervention activities: affective attitude, burden, ethicality, intervention coherence, opportunity costs, perceived effectiveness, and self-efficacy at completing intervention activities. A strength of this model is that it is based on a systematic review of reviews on measuring acceptability within health care interventions and thus is comprehensive in its scope. A limitation of this model is that some of the identified components overlap heavily with other components of feasibility, such as usability and practicality, thus we also report on some aspects of feasibility that are strongly related to acceptability [29,31]. Definitions of each component can be found in Table 1.

**Table 1:** Knowledge and Interpersonal Skills to Develop Enhanced Relationships (KINDER) codebook definitions.

Code	Definition
Acceptability	A multifaceted construct that reflects the extent to which people delivering or receiving an intervention consider it to be appropriate, based on anticipated or experiential cognitive and emotional responses to the intervention
Affective attitude	How an individual feels about the intervention
Intervention coherence	The extent to which the participant understands the intervention and how it works
Perceived effectiveness	The extent to which the intervention is perceived as likely to achieve its purpose
Burden	The perceived amount of effort that is required to participate in the intervention
Opportunity cost	The extent to which benefits, profits, or values must be given up to engage in the intervention
Self-efficacy	The participant’s confidence that they can perform the behaviors required to participate in the intervention

Generally, these components reflect participants’ perceptions of the intervention’s appropriateness based on their cognitive understanding of and emotional experiences during the intervention. Using a multicomponent framework of acceptability can uncover complex and nuanced responses to an intervention, allowing participants to endorse certain aspects while recognizing opportunities for improvement in others. Incorporating necessary adjustments identified in the findings from an acceptability study can improve intervention feasibility and overall acceptance and has driven efforts to continually improve the *KINDER* program [30].

### Data Collection

#### Survey Data

Quantitative data on intervention acceptability were collected through postintervention survey. Surveys were self-administered online using the Research Electronic Data Capture (REDCap) system and included 27 items that were developed for this study [32]. Items were broadly based on components of the TFA to address multiple aspects of acceptability and were iteratively developed through discussion with the investigator team, as a validated measure of acceptability using the TFA model does not yet exist [33]. Nevertheless, we found that TFA components were applicable to caregiver experiences in the first feasibility study that relied on qualitative interviews [14] and found that an author-generated acceptability measure based upon the model was preferable to other validated acceptability measures that are narrower in scope, especially given the ability to triangulate with qualitative interviews [34]. Reflecting overall acceptability, caregivers were asked about their overall satisfaction with *KINDER* and how likely they would be to recommend the program to other caregivers. Response options were recorded on a 5-point Likert scale, ranging from “Strongly disagree” (0) to “Strongly agree” (4). Reflecting the components’ “perceived effectiveness” and “opportunity costs” in the TFA model, caregivers also rated how valuable they found each component of the intervention to be, with response options including “Not at all valuable” (0), “Somewhat valuable” (1), and “Very valuable” (2). Detailed survey data about caregiver experiences while participating in the discussion sessions were collected, given these sessions were a newly added feature to the intervention. Caregivers rated their level of agreement with statements about the discussion sessions (eg, “I felt comfortable with sharing experiences with others”) on a 5-point Likert scale, ranging from “Strongly disagree” (0) to “Strongly agree” (4). Items evaluated multiple components of the TFA, such as perceived effectiveness, as well as “burden” given the time demands of attending an synchronous discussion session. Survey questions did not require a response. Reponses could be modified until the survey was submitted.

#### Interview Data

Purposive sampling was used to identify 23 caregivers enrolled in the feasibility study to participate in a one-time, in-depth, semistructured qualitative interview following participation in *KINDER.* Although this sampling technique risks selection bias, we used this approach to support the inclusion of perspectives of caregivers from various genders, races, ethnicities, and kin-relationships to the care recipient*.* Of the 23 caregivers invited, 11 agreed to participate. Initial interviews were conducted by the first author (KM), a female PhD faculty member with prior experience leading qualitative interview studies and who co-delivered the intervention. Later interviews were administered by an undergraduate student research assistant (SK) trained by the principal investigator who had no prior relationship with participants. All interviews were 1-on-1 with participants, except one interview where with both the principal and research assistant interviewers attended for training purposes. Interviews were held over Zoom videoconferencing software and video recorded for accuracy. The interview guide used to evaluate the acceptability of the revised *KINDER* intervention aligns with the one previously used to evaluate the initial version of the program, *KINDER* 1.0 (see Meyer et al [14]). Topics included caregiver perceptions on how their participation affected care relationships, the perceived skills developed while participating, the most and least valuable components, any potential discomfort with intervention modules, barriers to participation, and recommendations on how to improve the program. Interviews lasted an average of 37.8 (SD 21.2) minutes.

### Data Analysis

Survey data were analyzed using descriptive statistics, including frequencies, percentages, means, and measures of variance. Surveys were included even if responses to some items were missing. All analyses were conducted in Stata 18.0 [35]. Qualitative interview recordings were professionally transcribed before analysis. Transcripts were analyzed using a modified version of the thematic analytic approach by Braun and Clark [36]. This analytic approach was selected for its flexibility, including the ability to code both latent and manifest content. To establish credibility, a component from the criteria by Lincoln and Guba [37] to establish trustworthiness in qualitative research, transcripts were reviewed prior to applying the codebook [38]. Confirmability, another component of trustworthiness, was supported using a codebook that included clear definitions of each code [38]. Coding was primarily deductive and adhered to the 7 components of the TFA [30]. By using the TFA framework to inform survey questions, the codebook facilitated triangulation of method and study dependability. Two investigators, KM and WW, independently coded each interview using the codebook (see Table 1). Differences in coding were discussed until agreement was reached after each transcript was independently coded. The lead investigator reviewed excerpts under each code, identified key themes, and prepared a draft write-up of the findings. Data saturation was reached as coders identified no need to add new codes nor novel applications of codes. The resulting themes were validated by the second coder to ensure their accuracy, again supporting credibility in the analysis. All coding was conducted in NVivo 14 [39].

### Ethical Considerations

The trial was registered at ClinicalTrials.gov and received approval from the Case Western Reserve University Institutional Review Board (STUDY20221333). Additional protocol information is available on ClinicalTrials.gov. Caregivers enrolled in the feasibility study provided written consent to participate in this research. For caregivers who were not enrolled in the feasibility study, a study information sheet was provided at the beginning of the online survey. All qualitative interview participants received an information sheet prior to their scheduled interview and provided verbal consent at the start of the interview. Participants were notified of the voluntary nature and estimated length of study activities, of the name of the principal investigator, of the purpose of study activities including the investigators’ reasons for conducting interviews, and that their de-identified data would be retained indefinitely. Access to data was limited to IRB-approved study team members. Consolidated Criteria for Reporting Qualitative Research (COREQ) and Consolidated Standards of Reporting Trials (CONSORT) checklists are available in Multimedia Appendix 4 and Multimedia Appendix 5, respectively [40,41].

## Results

### Quantitative Findings

#### Sample Characteristics

Caregivers enrolled in the feasibility study (N=45) were, on average, 59 (SD 14.4) years of age (range: 34 to 87 years), and most indicated they were of the female sex (39/45, 89%). Slightly more than one-half (25/45, 57%) identified as white, followed by Asian (7/45, 16%) then “Other” or multiple races (5/45, 12%). Approximately one-third (15/45, 33%) of caregivers assisted a spouse, and a little over one-half (25/45, 56%) cared for a parent or parent-in-law. Most caregivers (33/45, 75%) had been in this role for at least 3 years. See the study by Meyer et al [15] for additional sample characteristics.

Satisfaction surveys were completed by a total of 71 of the 151 caregivers (47%) who registered to participate. In addition to the 37 caregivers (37/45, 82%) who completed the follow-up survey questions related to intervention acceptability as part of the feasibility study, another 34 caregivers who were not enrolled in the pre- and posttest study completed the satisfaction survey anonymously (34/53, 64%; see Multimedia Appendix 1 for additional information on completion rates). Demographic data are not available for these caregivers.

#### Survey Results

**Figure 1:**
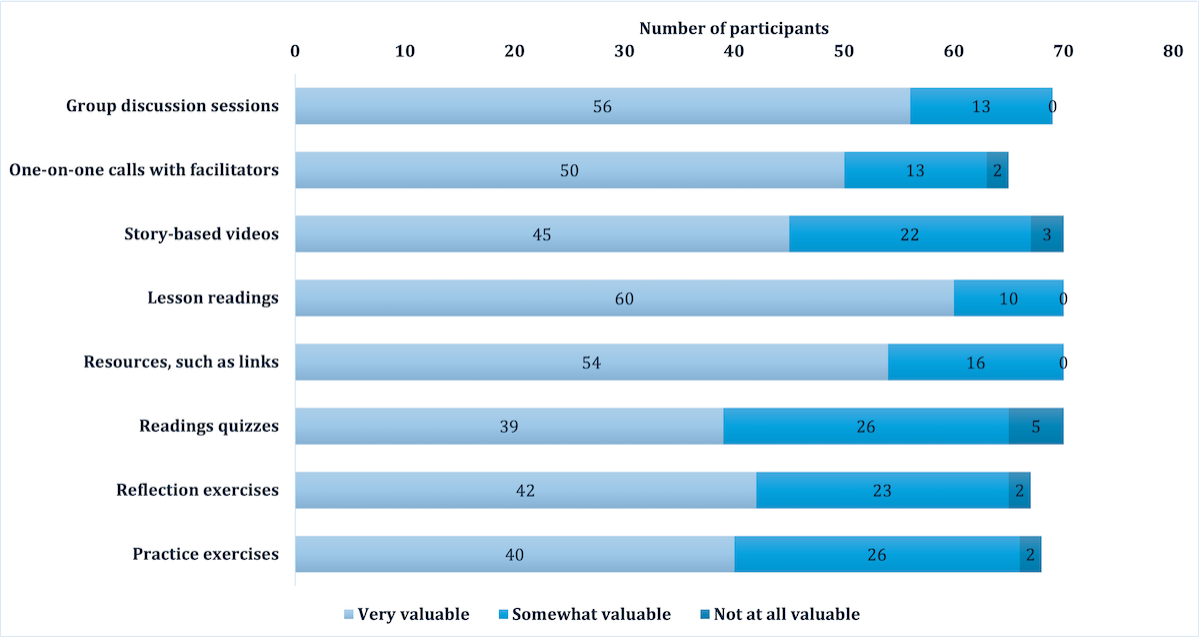
Satisfaction survey results on the perceived value of KINDER intervention components (N=71).

Caregivers reported high levels of overall satisfaction with *KINDER;* 80% (53/66) reported they were “Very Satisfied” with the intervention, and 20% (13/66) indicated they were “Satisfied.” When asked about their level of satisfaction with individual program components, most components were rated as being “Very valuable.” Group discussion sessions (56/69, 81%) and lesson readings (60/70, 86%) had the highest ratings, while the component most frequently rated as “Not valuable at all” was the reading quizzes given at the end of each independent lesson (5/70, 7%). The perceived value of all *KINDER* intervention components can be found in Figure 1. Group discussion sessions were also highly rated by caregivers, with most caregivers reporting they “Strongly agree” (46/68, 68%) or “Agree” (16/68, 24%) with the statement, “I enjoyed participating in group discussion.” See Table 2 for complete results about the level of agreement with the various features of group discussion sessions.

**Table 2:** Caregivers’ level of agreement with features of group discussion sessions (N=71).

Group discussion features	Strongly disagree, n (%)	Disagree, n (%)	Neither agree nor disagree, n (%)	Agree, n (%)	Strongly agree, n (%)
The facilitators were knowledgeable about the topics covered.^a^	0 (0)	0 (0)	1 (1)	8 (11)	61 (87)
I felt comfortable sharing my experiences with other caregivers.^a^	0 (0)	1 (1)	5 (7)	10 (14)	54 (77)
I learned new ideas from other caregivers.^a^	0 (0)	1 (1)	4 (6)	21 (30)	44 (63)
I enjoyed participating in group sessions.^b^	0 (0)	0 (0)	6 (9)	16 (23)	46 (68)

^a^Missing 1 case (n=70).

^b^Missing 3 cases (n=68).

### Qualitative Findings

#### Overview of the Themes

The thematic analysis revealed 4 core themes. The first theme focuses on caregivers’ overall attitudes toward *KINDER*, including its alignment with their values. The second theme pertains to caregivers’ beliefs about the effectiveness of the intervention at improving their caregiving relationships. The third theme captures the perceived difficulty with participating in the program relative to its perceived overall value. The final theme highlights opportunities the caregivers identified for refining future implementations of *KINDER*. Multimedia Appendix 2 presents the demographic characteristics of each caregiver. Multimedia Appendix 3 provides exemplars of each component of the TFA model by Sekhon et al [30]

#### Theme 1: Caregivers Reported a Positive Perception of the KINDER Intervention

The first theme characterizes caregivers’ overall attitudes toward *KINDER*, including the extent to which they enjoyed participating and understood the intervention’s purpose and how well the approach aligned with their values. Within the TFA model, this theme addresses the affective attitude, intervention coherence, and ethicality components of acceptability.

##### Caregivers Expressed a Positive Attitude Toward KINDER

Caregiver descriptions about their experiences with the intervention were overwhelmingly positive: **“**I loved the class,” said one caregiver (CG 45). Another shared, “I think this program should be shared the minute that somebody is diagnosed with something” (CG 12). Similarly, when closing out their interview, a participant caring for her husband expressed her wishes for *KINDER* to continue:

I will be praying that God will bless your program and help it to really help a lot of people to have a more positive relationship in caregiving.

CG 9

##### The Intervention Appeared Coherent, and Caregivers Understood its Intended Purpose

Caregivers understood that *KINDER* was developed “to promote a healthier relationship between the care recipient and the caregiver” (CG 35) and encourage a “kinder, gentler, more understanding approach [to caregiving]” (CG 15). They were also cognizant that *KINDER* focused on relationship quality “to increase the quality of care that the people—the care recipients—get” (CG 9). Although the intervention did not directly indicate that one of its goals was to prevent older adult mistreatment, caregivers were aware that part of improving the care relationship also included preventing negative interpersonal interactions. When asked about *KINDER*’s purpose, one caregiver helping her mother responded that it was to “prevent as much hostile or negative interaction that can easily arise in interacting with a loved one” (CG 42).

##### Caregivers Felt KINDER Aligned With Their Values

Despite tackling difficult topics, responses from participants suggest the intervention aligned with their values. “I feel very positive about the approach...I agree with the approach and how to handle situations,” said one participant (CG 35). Another caregiver particularly liked how the program emphasized that caregivers did not need to be “perfect” in their role. She felt that, in stressing this point, “it raised the standards of kindness, but it diminishes the standards of perfectionism” (CG 9).

Of particular interest to the investigators was whether the story-based videos depicting examples of relationship strain and potential mistreatment were consistent with caregivers’ values and preferences. Most participants were enthusiastic about these videos. They felt the videos were relevant: “I loved the video material. I thought that was super helpful and you could relate” (CG 45). One caregiver even expressed that she could see herself in the videos: “So, that week seven video could’ve been me. The kitchen one. I do remember that” (CG 27). Although most caregivers felt that the videos were tasteful in their approach, a few caregivers expressed that the videos were a “little over the top” (CG 22). One participant remembered thinking to herself: “‘Really? People can go there?’” (CG 12). Moreover, although there was awareness that the videos could be uncomfortable to watch, there was a general feeling that their value was worth some potential discomfort. The same participant who expressed initial surprise that caregivers “can go there” later recognized their worth during her interview, saying that the videos “remind you that you can go to a place if you’re not ready, and I think by being ready you can avoid going there” (CG 12).

#### Theme 2: Caregivers Described the Intervention as Beneficial to Their Care Relationship

Participants perceived that *KINDER* helped them address their caregiving relationship and improved the quality of caregiving. Within the TFA model, this theme primarily relates to the “perceived effectiveness” component.

##### KINDER Facilitated a Perceived Reduction in Relationship Strain

Specifically, caregivers reported that *KINDER* helped them reframe their interactions with the care recipient, enabling them to better understand their behaviors and respond more appropriately:

So, really the KINDER program just really opened my eyes to the fact that I wasn’t seeing him as he is today. I was reacting to him from old historical references instead of what’s happening present day right here now.

CG 35

One caregiver, currently caring for his wife, reflected on how participating in the program would have benefited him when he cared for his late father. He described feeling anger toward his father and wondered whether attending a course like *KINDER* might have influenced his earlier approach to caregiving:

I was trying to be very helpful, but I was also quite angry with him. And, um, had I taken a course like this or been exposed in some way to, um, more effective caregiving, I think I would have approached him very differently.

CG 15

##### Caregivers Gained Skills to Help Manage Relationship Strain and Improve Care Quality

Several participants provided specific examples of how they applied the intervention’s lessons to modify their caregiving approach. For example, one of the strategies taught in *KINDER* to reduce relationship strain is to “go with the flow.” One caregiver helping her mother described how she has applied this mantra:

I used to think, my mom has to listen to me, and that’s the way it’s supposed to be. Now, I’m more relaxed. That’s the way. I will go with the flow.

CG 19

Participants also applied other skills to reduce relationship tension with the care recipient, such as redirecting the care recipient’s attention to manage the behavioral symptoms of dementia or cognitively reframing the situation:

Learning all those skills from the book and then, kinda like how to divert or think of positive side instead of drilling on the negative side of why I am taking care of this person. I think it’s really helpful in building relationship with my parent.

CG 14

Similarly, another participant recognized the need to modify her interactions with her husband in response to changes in his cognition:

You know I said, “Oh, wait a minute, I’ve got to, you know, tone it down a little bit,” and just [9] a different approach, and just some reminders about of all of that.

CG 12

Perhaps most interesting was the caregiver who shared a reflection exercise response, where they applied intervention content about intimidating body language to their care situation:

My week seven reflection was basically, I just wrote something very short, I wrote “Physical aggression that doesn’t involve hitting, but that can be just as affecting. It causes more confusion and is likely more isolating. Even though she is very tough, it’s not nice to make things scarier than they need to be for her, ever.”

CG 27

This reflection reinforced an important message about relationship strain and psychological mistreatment from the intervention, highlighting where caregivers’ body language can be a source of intimidation.

#### Theme 3: Caregivers Felt Participating in KINDER Was Worthwhile

The third theme explores whether the caregivers felt the time and effort required to participate in *KINDER* was justified. This theme aligns with the burden, opportunity costs, and self-efficacy components within the TFA model. Overall, caregivers reported participation in *KINDER* was worthwhile and not overly burdensome.

##### Caregivers Reported That KINDER Was “a Very Good Use of Time” (CG 15)

When asked whether there was content that should be removed or condensed, none of the caregivers had any suggestions:

I didn’t really find anything that was just like so left field that I was like, “Oh, I don’t need this.” Not at all. I felt like every reflection, I had something to write and say.

CG 27

Echoing this statement, another caregiver said:

I think all of the chapters [of the workbook] are very helpful. I think the whole thing should be kept.

CG 14

Even one initially skeptical participant eventually came around to acknowledging that *KINDER* was worthwhile:

And I think when it was first going on, I was wondering, “What? Do I really need to be involved in this kind of stuff?” And the answer was of course. Resoundingly so. And so, I’m feeling, I would say, a lot closer to [care recipient name] now than I was three or four years ago.

CG 15

##### Caregivers Found That Participation Was Not Overly Burdensome

When caregivers reported any difficulty in participating, they felt that any burden imposed by the intervention was worth the effort. For example, some caregivers felt the independent lesson readings in the workbook could be overwhelming:

I will say it was hard to make the time to do the reading. And that’s interesting because I was not in the total thick of it. If I had had to try to do that reading and watch the videos while I was in the total thick of it, I don’t know if I would have been able to do it.

CG 42

On the other hand, sharing enthusiasm for workbook content, another caregiver said, “I’m never throwing this book away” (CG 29). Caregivers appreciated the email reminders sent each week to help keep them on track with readings and practice exercises:

I love your reminder every week to do your homework. That’s a good reminder because we’re so busy with other things.

CG 19

Caregivers reported that the regular reminders and the discussion meetings created a sense of “accountability,” where, as one caregiver described it, “you’re [39] just sort of on an island, reading a book. It’s like, no, you’re in touch with people that are inquiring as to these things” (CG 27).

#### Theme 4: Caregivers Identified Opportunities to Improve KINDER

The fourth theme describes insights caregivers offered about how *KINDER* could be adapted to better meet their needs. Although participants generally found the program worthwhile, interviews identified opportunities for improvement, particularly around increasing caregiver interaction and making participation more convenient. The following recommendations highlight key areas for improving the program’s accessibility and engagement.

##### Recommendation 1: Provide Additional Opportunities for Caregivers to Connect

The primary recommendation to improve *KINDER*’s acceptability was to provide more opportunities for caregivers to connect with other cohort members. Several caregivers suggested increasing the number of group discussion sessions. “I think you should have more online meetings with everybody where they can discuss things,” shared one caregiver (CG 22). Another participant felt that meeting once per week may have been more beneficial than the 3 meetings spread throughout the 8 weeks: “I feel like maybe once a week would be a better way to gain insight from others going through the same or similar situation” (CG 35).

##### Recommendation 2: Facilitate Opportunities for Interaction During the Discussion Sessions

Despite an overall appreciation for the group sessions, some caregivers felt sessions could have been more interactive. One caregiver shared that the groups “need more interaction between each other somehow, or a little group breakout room” (CG 27). Other ideas included encouraging caregivers to turn on their cameras during the group sessions and incorporating more icebreakers to help participants get to know each other better. One participant offered insights on improving session facilitation to promote more exchange, noting that when a single caregiver’s concern took up a large part of the group session, it negatively affected their experience:

I feel for them and I’m sorry that they’re experiencing that...But I do feel like that did go on a little bit long.

CG 45

##### Recommendation 3: Integrate Support to Make Participation More Convenient

Caregivers proposed ways to make participation more convenient. Some suggested sending text message reminders, in addition to emails, for independent lessons and discussion sessions: “I don’t get on my email as much, but my text [messages]—I always have my phone” (CG 12). To make it easier to participate in independent lessons, another idea was to audio record the assigned readings so that caregivers could listen to lessons while doing other activities such as “during their commute” (CG 29). Another caregiver suggested delivering the program via an app or web-based platform where they could complete lesson activities online:

There needs to be just look at it on your phone...There’s click here to enter it to keep a log, to use the website or whatever, to save your work.

CG 42

When asked about how to reconcile different needs and preferences of caregivers regarding the integration of technologies, like an app or website, one participant responded that the best solution was to offer choices and used a wheelchair ramp as an analogy:

People think, “Oh, let’s see what the majority will use.” Who gives a crap? If one person needs a wheelchair ramp, we need a wheelchair ramp.

CG 42

## Discussion

### Summary of Findings

This study aimed to assess the acceptability of the latest version of an intervention to prevent psychological mistreatment by family caregivers to persons with dementia. We are unaware of any other study that has reported on the acceptability of caregiver interventions developed to prevent older adult mistreatment, making this work a novel addition to the current literature. Findings support *KINDER*’s overall acceptability among caregivers to persons with dementia. Postintervention satisfaction survey responses reflect that caregivers would recommend the intervention to other caregivers and that caregivers found each component of the intervention valuable. One of the primary changes between the current version of *KINDER* and the previous version is the addition of 3 virtual group discussion sessions. Survey data show that caregivers had a positive experience with these discussion groups. Qualitative data support this finding, revealing that some caregivers would even be interested in additional group discussions throughout the 8-week program. By using multiple methods of data collection, this study offers a holistic understanding of how *KINDER* was perceived by caregiver participants. In so doing, findings not only support the intervention’s acceptability but also highlight opportunities to continue improving caregivers’ experiences during future program implementations.

Findings, particularly those from the qualitative interviews, demonstrate a positive affective attitude toward the program, alignment with values (ethicality), and the overall belief that the demands of participating were not too high and mostly worthwhile (opportunity cost and burden). These findings align with those of the initial first web-based, asynchronous version of *KINDER* 1.0, where participants also completed qualitative interviews following the intervention [14]. Whereas the earlier study applied the TFA after conducting thematic analysis, this study integrated components of the framework into the coding process to more clearly evaluate their application to caregiver experiences with *KINDER* more closely [30].

One of *KINDER*’s strengths identified in both studies was the inclusion of story-based videos. Videos and visual examples have been found to enhance the experience of caregivers participating in interventions [31]. Prior studies of digital caregiver interventions have found that participants value videos portraying the “messiness” of caregiving [42]. A comparison of qualitative findings from the two feasibility studies of *KINDER* suggests that challenges related to participation were largely addressed. For example, in the earlier study (*KINDER* 1.0), caregivers reported technological issues affected their experiences. After simplifying the mode of delivery from a web-based delivery platform to a PDF or print workbook, qualitative interview data collected in the second study showed far fewer concerns about technology being a barrier to participation than in the first study. This suggests technology issues may have been mitigated with the modifications made to *KINDER* in the second study. Nevertheless, this concern still requires ongoing consideration in future studies, as nonresponders for interviews and surveys may have had different experiences than responders. Another obstacle reported by caregivers who participated in *KINDER* 1.0 was that the content could be overwhelming and they lacked time to complete independent lessons. However, fewer caregivers in the revised *KINDER* program identified this as a challenge*,* likely due to its regular check-in reminders, group discussion sessions, and flexible scheduling that allowed participants to “catch up” on independent learning activities when needed. Furthermore, we also found that group-based sessions recommended by caregivers in the earlier study enhanced participant experiences in the *KINDER* intervention. Caregivers, for the most part, felt connected to others in their cohort. Some even wished for more opportunities to connect. This finding is consistent with those from similar caregiver interventions that use a hybrid group-based and asynchronous delivery approach [42,43].

### Comparison With Other Interventions to Support Caregiving Relationship Quality

There are several interventions focused on improving caregiving relationships, though not necessarily on prevention of older adult mistreatment, providing points of comparison with our findings. In the *At* *The Crossroads* caregiver intervention, researchers conducted a randomized controlled trial of a psychoeducational intervention for driving cessation among persons with dementia [44]. Like *KINDER*, this intervention emphasized the potential for varying priorities between the caregiver and person with dementia, particularly related to the balance of safety and autonomy. Findings revealed that caregivers in the intervention group felt more prepared to discuss driving cessation with their family member and were less worried about upsetting or angering the care recipient than those in the control condition. This aligns with findings from our study, wherein caregiver participants similarly felt more prepared to reframe interactions with the care recipient and reported enacting skills taught in the intervention to promote positive care relationships. Although the two interventions—*At The Crossroads* and *KINDER*—have distinctly different overall goals (driving cessation versus preventing older adult mistreatment), both interventions leverage existing interpersonal relationships to improve relational functioning through similar approaches.

Another intervention well-suited for comparison is the *Support, Health, Activities, Resources, and Education* (*SHARE*) intervention. This intervention is designed to reconcile care values between caregivers and those with dementia while creating a plan for future care based on these shared values [45]. Unlike *At a Crossroads* and *KINDER*, *SHARE* is an in-person intervention that includes both care partners and a trained counselor. However, like these other interventions, *SHARE* was delivered over 6 weekly sessions and explicitly addresses the reconciliation of care values, including safety and autonomy. Caregivers participating in *SHARE* reported satisfaction with the overall experience of participating, the counselor, the weekly sessions, and their relationship functioning with the care recipient postintervention [45]. Qualitative findings also affirm overall satisfaction, as well as the application of new skills, development of knowledge, and greater awareness of available resources [46]. Caregivers in *SHARE* reported similar challenges to those identified in the evaluation of *KINDER* 1.0, such as challenges with finding time to participate and difficulties navigating emotionally intense content. These similarities suggest that interventionists should be mindful of the potential for emotional distress among participants.

### Opportunities to Improve the Current KINDER Intervention

Findings from this study indicate opportunities to improve *KINDER* and enhance intervention acceptability. First, caregivers expressed an interest in using technology to enhance convenience when participating, suggesting text message reminders in addition to email. This recommendation holds promise in terms of likely acceptability and feasibility, given that 82% of caregivers use a smartphone and recent research shows that caregivers find text-message delivered interventions to be highly acceptable [47,48]. However, although text messaging is a relatively simple technology integration, caution is needed when considering more complex digital intervention delivery models where the time and effort required for participants to learn these systems may outweigh their perceived benefits [31]. *KINDER* is unique in that its first iteration took place via a web-based portal, before it was revised to make it more accessible and easier for caregivers to participate. This raises a dilemma: To what extent should caregiver preferences for integration of digital technologies be heeded when prior applications demonstrate a lack of engagement?

Multiple factors should be considered when addressing this question and when interpreting study results. First, the context of intervention delivery for caregivers has changed tremendously in recent years and may offer an alternative explanation as to why technology barriers were featured less often in the second feasibility study than the first one. Social distancing restrictions put in place during the COVID-19 pandemic accelerated the transition to digitally delivered programming, which may have increased caregivers’ comfort with digital platforms [49]. Although the pandemic most likely accelerated acceptance and uptake of digital caregiver interventions given service organizations’ efforts to provide technology support [50,51], this shift likely occurred within the broader context of already evolving social and cultural norms around technology adoption as society continues to acclimatize to emerging technologies [16]. If the online version of *KINDER* (*KINDER* 1.0) was offered today, it is plausible that it would achieve greater acceptance than when it was initially offered from 2020 to 2021.

Another consideration was illustrated by a study participant who revealed the importance of accommodating varying needs and preferences for intervention delivery: “If one person needs a wheelchair ramp, we need a wheelchair ramp.” This statement aligns with calls to prioritize health equity when developing and testing digital solutions [52]. Based on our findings, we propose that, rather than optimizing an intervention’s delivery approach to fit the needs of most caregivers, interventions should instead be offered in multiple formats to accommodate varying needs. This aligns with prior recommendations to offer digital materials in a variety of formats to meet different needs [53]. Beyond offering options for participation, in future studies, we intend to measure digital literacy among users to better distinguish between the acceptability of a digital intervention and users’ level of digital literacy. Limited digital literacy can influence user acceptability and indicates a need for strategies targeted at digital literacy specifically to improve acceptability (eg, technology coaching to increase comfort with digital tools) [54]. Further, during intervention eligibility screening, we will identify the frequency at which limited access to digital devices and internet prevents participation. A recent scoping review identified inequitable internet access as a structural barrier to caregivers accessing community support services [55]. Overcoming inequitable broadband access is also a recommendation in the United States’ 2022 National Strategy on Caregiving to enable easier access to digital caregiver supports [56].

Further, although caregivers expressed an interest in having more opportunities to connect with group members, some participants in the *KINDER* program voiced concerns about the perceived low level of engagement from others. For example, one caregiver suggested the facilitators encourage participants to turn on their cameras during group sessions so others could see their faces, and another suggested adding more icebreakers to kick off the group sessions. In their qualitative study of the *TeleSavvy* intervention, Kovaleva et al [42] identified a similar issue, where participants expressed bother when they noticed other participants were distracted. Low engagement among certain participants can undermine the experience of others, who may benefit less from group discussion and shared examples from others, making this a serious concern. At the same time, behaviors that may appear as disinterest could be driven by factors related to health equity, such as lacking additional support that would allow a caregiver to participate in *KINDER* without disruption. Indeed, one caregiver in this study described having to listen to one of the discussion sessions while picking up her daughter from school, rendering her unable to contribute to group conversations. We partially addressed this in *KINDER* by encouraging caregivers to keep their cameras on during sessions, while also reminding them that it is okay to go “off camera” when needed. Still, given the variability this creates for participants between cohorts, further consideration may be needed to understand how to address participant engagement in synchronous group-based caregiver interventions. Nevertheless, observer fidelity reports indicate that in 17 of 18 sessions where participant engagement in group sessions was assessed, observers “Agreed” or “Strongly agreed” that participants were engaged [15].

### Limitations

This study has limitations. First, the initial qualitative interviews were led by the lead investigator, who also cofacilitated the intervention. As a result, participants’ responses may have been affected by a social desirability bias. However, there was no apparent difference in participant attitudes in interviews conducted with another member of the research team and those who spoke with the lead investigator. In both instances, caregivers provided both criticism and praise of the *KINDER* program. Rapport with the lead investigator may also have facilitated trust so that participants felt more comfortable sharing negative feedback and how they applied what they learned to manage challenging care relationships [57]. Although sample representativeness is not an objective of qualitative studies, it is possible that results were affected by self-selection bias where caregivers who had more positive experiences agreed to be interviewed.

Another limitation is that this study conflated the concepts of usability and acceptability, recognized in the literature as distinct concepts. Although we applied the validated TFA to help evaluate our intervention’s acceptability among caregivers, future studies should also include standardized measures of acceptability and usability that delineate these interconnected though distinct concepts to better inform intervention refinement [16,31]. Nevertheless, we used an established acceptability framework (TFA) to guide the qualitative interview data collection and analysis, which enabled us to evaluate various dimensions of intervention acceptability. At the time when this study was administered, there was no validated questionnaire available based on the TFA. Such a questionnaire is under development and will be a valuable tool to measure *KINDER*’s acceptability once validated. The use of a nonvalidated measure in this study weakens the generalizability of our quantitative findings and can undermine future replication efforts. Finally, demographic data were not available from participants who completed the satisfaction survey. Nevertheless, data from the online event registration form revealed no statistically significant difference in age, gender, race, ethnicity, educational attainment, nor financial difficulty of caregivers in the study compared with those registered to attend without being in the study [15].

### Conclusion

This study advances current knowledge on interventions for caregivers of persons with dementia to prevent older adult mistreatment by demonstrating the acceptability of a novel intervention, *KINDER*. Additional research is needed to test the efficacy of the intervention, including the plausibility of theoretical mechanisms that may drive a reduction of psychological EM, and to continue evaluating the implementation of the intervention within health and social service settings. Specifically, the next step for this research will involve conducting a fully powered randomized controlled trial as part of a Stage II efficacy trial in a controlled research setting [58]; in addition to measures of study outcomes, study participants randomized into the intervention arm will be asked about intervention acceptability in a postintervention survey to ensure ongoing acceptability. To prepare for effectiveness testing in applied settings, such as Area Agencies on Aging, we will interview service providers to identify how the *KINDER* intervention may need to be adapted to fit provider workflows and organizational processes [59]. Nevertheless, by using quantitative and qualitative methods to assess multiple components of acceptability, this study provides a robust picture of the acceptability of one of the few interventions designed to prevent EM in a family care context. If proven efficacious, the *KINDER* intervention has the potential to promote care that upholds the dignity and safety of both caregivers and persons with dementia [15]. However, this potential can only be met if the intervention is acceptable to users. Findings from this study indicate that KINDER’s intended audience, family caregivers, find the intervention to be acceptable, though ongoing evaluation of acceptability of KINDER and similar programs is needed.

## Appendix

Multimedia Appendix 1 Creation of analytic sample for satisfaction surveys from program registrants.

Multimedia Appendix 2 Demographic information for qualitative interviews (N = 11).

Multimedia Appendix 3 Exemplars of each component of acceptability from the Theoretical Framework of Acceptability (TFA) model by Sekhon et al.

Multimedia Appendix 4 COREQ checklist.

Multimedia Appendix 5 CONSORT 2010 checklist.

## Abbreviations

APS: Adult Protective Service
CONSORT: Consolidated Standards of Reporting Trials
COREQ: Consolidated Criteria for Reporting Qualitative Research
KINDER: Knowledge and Interpersonal Skills to Develop Enhanced Relationships
REDCap: Research Electronic Data Capture
TFA: Theoretical Framework of Acceptability
WHO: World Health Organization

## Data Availability

Data from both studies described in this manuscript are not available due to the sensitive nature of topics discussed with participants.
