# Supplementary material for: Acceptability of an Intervention to Prevent Older Adult Mistreatment Among Family Caregivers to Persons With Dementia: Multimethod Pilot Study
Source: JMIR Form Res. 2025 Jul 30;9:e73778. doi: 10.2196/73778 (PMC12351192; doi:10.2196/73778)
Supplement: Multimedia Appendix 1 [file formative_v9i1e73778_app1.docx]

### Multimedia Appendix 1: Creation of analytic sample for satisfaction surveys from program registrants


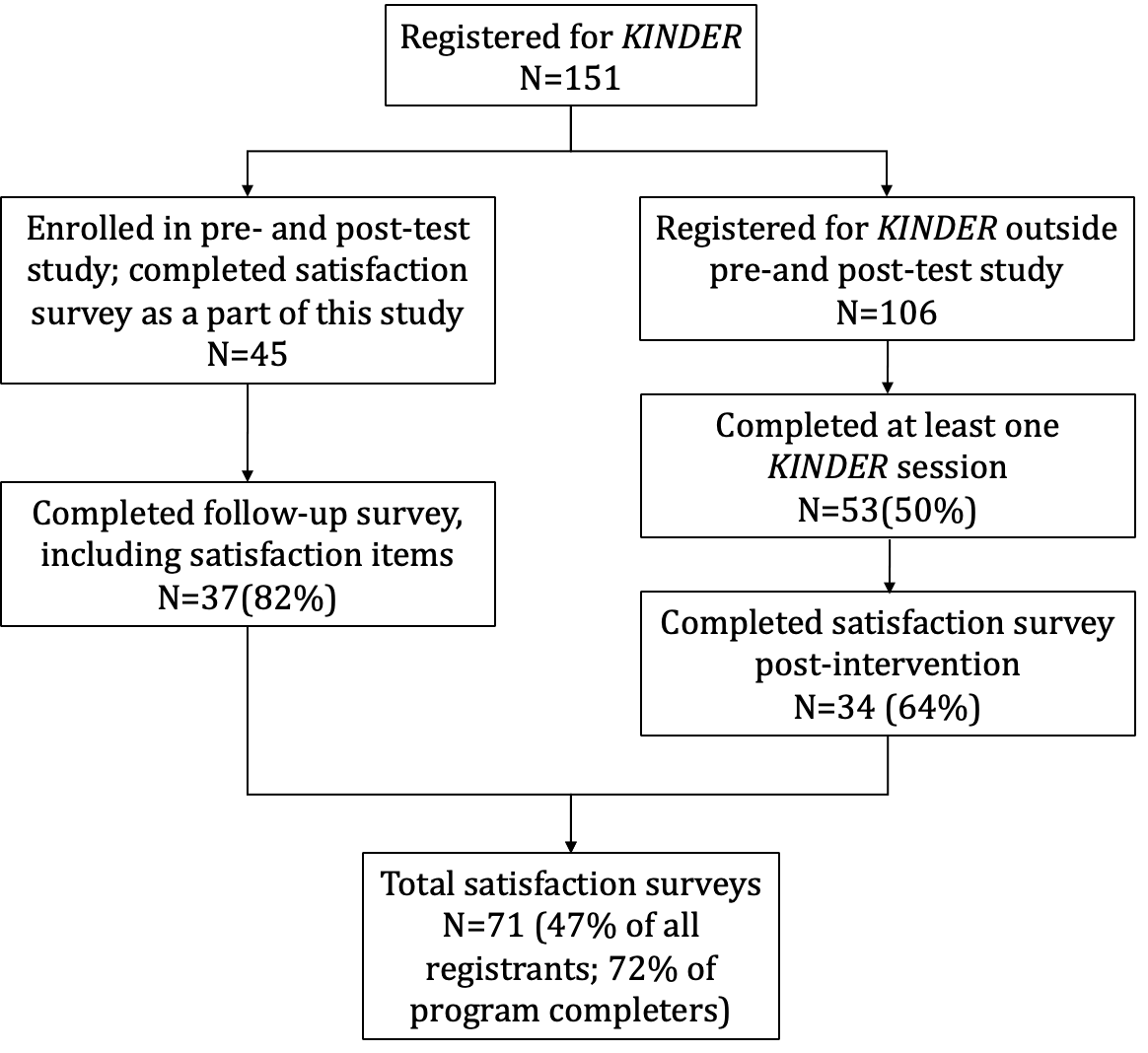


**Differences in survey administration.** There were minor differences in how surveys were administered for caregivers enrolled in the pre- and post-test study, and those who completed the *KINDER* program without being enrolled in the feasibility study. These are summarized below.

**Participant incentives:** Caregivers enrolled in the feasibility study received up to $150 in Amazon gift cards to participant in study activities, including the satisfaction survey, and could earn another $25 gift card for completing a qualitative interview. Satisfaction survey items were included in a survey that included 130 items, in addition to satisfaction questions. Participants who were not enrolled in the study did not receive payment for completing the satisfaction survey.

**Survey invitations:** Participants enrolled in the pre- and post-test study received an email with a unique URL inviting them to complete satisfaction items as a part of their follow up survey, such that duplicate responses from the same email could not be entered. This email was sent within 48 hours of intervention completion. These caregivers received a reminder every 2 days to complete the survey until it was completed and received up to 3 reminders. Caregivers who were not enrolled in the study were emailed using the address provided at registration. These caregivers received an initial email sent within 48 hours of completing the program, and a reminder email one week later.
