# Supplementary material for: Acceptability of an Intervention to Prevent Older Adult Mistreatment Among Family Caregivers to Persons With Dementia: Multimethod Pilot Study
Source: JMIR Form Res. 2025 Jul 30;9:e73778. doi: 10.2196/73778 (PMC12351192; doi:10.2196/73778)
Supplement: Multimedia Appendix 2 [file formative_v9i1e73778_app2.docx]

### **Multimedia Appendix 1: Demographic information for qualitative interviews (N = 11)**

| **Length of Caregiving** | More than 5 years | 3 to 5 years | 1 to 2 years | 3 to 5 years | More than 5 years | More than 5 years | 3 to 5 years | More than 5 years | 3 to 5 years | 3 to 5 years | More than 5 years |
| --- | --- | --- | --- | --- | --- | --- | --- | --- | --- | --- | --- |
| **Care Recipient Relationship to Caregiver** | Husband | Husband | Father (including step and in-law) | Wife | Mother | Wife | Other relative | Mother (including step and in-law) | Other relative | Mother (including step and in-law) | Husband |
| **Highest Level of Education** | Bachelor’s degree or Higher | Bachelor’s Degree or Higher | Bachelor’s Degree or Higher | Bachelor’s degree or Higher | Bachelor’s degree or Higher | Bachelor’s Degree or Higher | Some College | Vocational Training | Associate’s degree | Bachelor’s degree or Higher | Some College |
| **Ethnicity** | Not Hispanic or Latino | Hispanic or Latino | Not Hispanic or Latino | Not Hispanic or Latino | Not Hispanic or Latino | Not Hispanic or Latino | Not Hispanic or Latino | Hispanic or Latino | Hispanic or Latino | Not Hispanic or Latino | Hispanic or Latino |
| **Race** | White | White | Asian | White | Asian | More than one race | Other | Other | More than one race | White | White |
| **Gender** | Woman | Woman | Woman | Man | Woman | Man | Woman | Woman | Woman | Woman | Woman |
| **Age** | 75 | 63 | 43 | 77 | 79 | 75 | 46 | 46 | 67 | 47 | 53 |
| **Case** | 9 | 12 | 14 | 15 | 19 | 22 | 27 | 29 | 35 | 42 | 45 |
