# Supplementary material for: Acceptability of an Intervention to Prevent Older Adult Mistreatment Among Family Caregivers to Persons With Dementia: Multimethod Pilot Study
Source: JMIR Form Res. 2025 Jul 30;9:e73778. doi: 10.2196/73778 (PMC12351192; doi:10.2196/73778)
Supplement: Multimedia Appendix 3 [file formative_v9i1e73778_app3.docx]

### Multimedia Appendix 3: Exemplars of each component of acceptability from the Sekhon TFA Model.

| **Component** | **Exemplar** |
| --- | --- |
| **Acceptability** | *I think this program should be shared the minute that somebody is diagnosed with something. The doctor should say, 'Hey. Go to this.' Because I wish I would have had this way in the beginning.* (CG 12) |
| **Affective attitude** | *I think that overall, I really loved how the program was – I felt – it gave a very honest picture of caregiving*. (CG 45) |
| **Intervention coherence** | Responding to question about the purpose of *KINDER*: *To promote, like you said, a healthier relationship between the care recipient and the caregiver. And also, I think there was a lot of emphasis on taking care of yourself as a caregiver in order to give better care to the recipient.* (CG 35) |
| **Perceived effectiveness** | *Learning all those, like, skill from the book and then, um, kinda like how to, uh, divert or think of positive side instead of, like, uh, drilling on the negative side of why I am taking care of this person. Um, I think, um, it really helpful in building relationship with my parent. Um, it helped me to, um, learn, um, how to see silver lining in the situation*. (CG 14) |
| **Burden** | *The only thing that I saw, for me, personally, it would’ve been a little bit easier if the meetings were in the evening. . . That’s why I missed the last one because it was – I work during the day.* (CG 29) |
| **Opportunity cost** | *And then, overall, as I think back at it, I would say it was a very good use of my time. One can’t be reminded enough of things that fall into this category. I mean, we’re talking about the lives and wellbeing of our loved ones*. (CG 15) |
| **Self-efficacy** | *And the other thing is the lessons, because there was one week I didn't do it. So, the second week, I forgot which lesson, I doubled it. So, and I don't feel bad. I says, “I can catch up. It's okay.” So, this structure is real good.* (CG 19) |
